# Phosphorus-Use-Efficiency Gene Identification in Fabaceae and *RSL2* Expansion in *Lupinus albus* Is Associated with Low-Phosphorus Adaptation

**DOI:** 10.3390/genes15081049

**Published:** 2024-08-09

**Authors:** Xing Li, Jinyong Yang, Qian Zhang, Lingkui Zhang, Feng Cheng, Weifeng Xu

**Affiliations:** 1 Joint International Research Laboratory of Water and Nutrient in Crop, Fujian Provincial Key Laboratory of Plant Functional Biology and College of Resources and Environment, College of JunCao Science and Ecology, Fujian Agriculture and Forestry University, Fuzhou 350002, China; xzdlixing@126.com (X.L.); 200525003@fafu.edu.cn (J.Y.); qian_z@fafu.edu.cn (Q.Z.); 2 State Key Laboratory of Vegetable Biobreeding, Key Laboratory of Biology and Genetic Improvement of Horticultural Crops of the Ministry of Agriculture and Rural Affairs, Sino-Dutch Joint Laboratory of Horticultural Genomics, Institute of Vegetables and Flowers, Chinese Academy of Agricultural Sciences, 12 Zhongguancun South Street, Beijing 100081, China; zhanglk960127@163.com

**Keywords:** Fabaceae, white lupin, phosphorus utilization efficiency, cluster root, *RSL2*

## Abstract

Phosphorus is critical for plant growth but often becomes less accessible due to its precipitation with cations in soil. Fabaceae, a diverse plant family, exhibits robust adaptability and includes species like *Lupinus albus*, known for its efficient phosphorus utilization via cluster roots. Here, we systematically identified phosphorus-utilization-efficiency (PUE) gene families across 35 Fabaceae species, highlighting significant gene amplification in PUE pathways in Fabaceae. Different PUE pathways exhibited variable amplification, evolution, and retention patterns among various Fabaceae crops. Additionally, the number of homologous genes of the root hair development gene *RSL2* in *L. albus* was far more than that in other Fabaceae species. Multiple copies of the *RSL2* gene were amplified and retained in *L. albus* after whole genome triplication. The gene structure and motifs specifically retained in *L. albus* were different from homologous genes in other plants. Combining transcriptome analysis under low-phosphorus treatment, it was found that most of the homologous genes of *RSL2* in *L. albus* showed high expression in the cluster roots, suggesting that the *RSL2* gene family plays an important role in the adaptation process of *L. albus* to low-phosphorus environments and the formation of cluster roots.

## 1. Introduction

Fabaceae is one of the most important families in plant ecosystems, including 765 genera and approximately 19,500 species [1]. The number of Fabaceae species ranks third among angiosperms, following Asteraceae and Orchidaceae. The morphological and habit diversity of Fabaceae plants varies greatly, with distributions ranging from mountains, forests, and grasslands to plains, waters, and even deserts. The diversity of their habitats also reflects the strong adaptability of Fabaceae plants to different environments, making them one of the most successful evolutionary groups in angiosperms.

All existing angiosperms have undergone polyploidization events, which refer to the process of genome duplication [2,3]. Following polyploidization, all genes within the genome are replicated, and these replicated genes accumulate mutations and may develop new functions during the evolutionary process [4]. Previous studies have found that following the diversification of Fabaceae plants, many gene families exhibit specific distributions among different species [5]. For example, systematic analysis of the *Cercis chinensis* and multiple Fabaceae genomes discovered that newly emerged genes exhibit significant differences from pre-existing genes in terms of the exon length, exon number, and expression patterns [6]. This provides a foundation for biological evolution and serves as an accelerator for the evolution of gene functions. Fabaceae plants have all undergone whole-genome duplication events, which may play a crucial role in their efficient utilization of soil nutrients [7,8,9,10,11,12,13,14]. 

Phosphorus plays a pivotal role in plant growth, necessitating regular applications of phosphorus-based fertilizers to ensure sustainable crop yields [15]. Plants primarily take up phosphorus in the form of soluble inorganic orthophosphate (Pi), which exhibits significantly lower mobility in soil compared to most other nutrients, whereas Pi has a tendency to readily bind to mineral surfaces or precipitate with cations, such as aluminum (Al), iron (Fe), and calcium (Ca), resulting in a decrease in its solubility and diffusion capacity [16,17]. During the evolutionary process, plants have developed numerous adaptive mechanisms to acquire phosphorus from the soil [18,19]. Some plants adapt to low phosphorus by increasing root hair elongation and density [20,21]. 

White lupin (*L. albus*, 2n = 50), a member of the Fabaceae family, has evolved from a whole-genome triplication (WGT) event [22]. It possesses the ability to enhance the mobilization of soil phosphates through the development of tightly clustered lateral roots, also known as cluster roots or proteoid roots. Researchers have extensively investigated the morphological and physiological mechanisms behind its adaptation to low-phosphorus environments [23]. Nevertheless, the intricate interplay between the genome evolution and this specific low-phosphorus adaptation in white lupin remains unclear.

The genes responsible for encoding ROOT HAIR DEFECTIVE SIX-LIKE (RSL) proteins play a crucial role in positively modulating the development of root hair cells in diverse plant species, including *Arabidopsis thaliana*, *Oryza sativa*, and *Brachypodium distachyon* [24,25,26,27,28,29]. It was reported that enhanced expression of *RSL* genes in rice, Arabidopsis, and wheat led to an increased root hair length [29,30,31]. It was also reported that increasing root hair length through breeding or biotechnology can improve phosphate-uptake efficiency in Brachypodium [16]. 

However, the gene characteristics for high-efficiency phosphorus utilization in white lupin remain unclear. In this study, we performed phylogenetic analysis using 35 publicly available Fabaceae genomes. Through a systematic analysis of 35 Fabaceae genomes, we discovered the remarkable amplification of PUE-related gene families compared to *A. thaliana*. Further research revealed diverse patterns of amplification, evolution, and retention in PUE pathways among different Fabaceae crops, highlighting the complexity and diversity in phosphorus utilization among these plants. Notably, the *RSL2* gene family in white lupin, crucial for root hair development, was found to have significantly more copies than in other Fabaceae species. Multiple copies of the *RSL2* gene were amplified and retained in white lupin after WGT. The specific gene structures and motifs retained in white lupin differ from other homologous genes. When combined with transcriptome analysis, it was evident that most *RSL2* homologs in white lupin showed high expression in cluster roots under low-phosphorus conditions, indicating a crucial role in the adaptation to phosphorus-deficient environments and the formation of cluster roots for white lupin.

## 2. Materials and Methods

### 2.1. Downloading the Published Genomes of Fabaceae Crops

The website https://www.plabipd.de/plant_genomes_pa.ep (accessed on 8 June 2023) was used to search for published Fabaceae plant genomes, and the NCBI at https://www.ncbi.nlm.nih.gov (accessed on 20 June 2023) was used to download the genome and gene annotation files.

### 2.2. Analysis of Evolutionary Relationships of Fabaceae Crops

In this study, we extracted protein sequences of genes from 35 downloaded Fabaceae plant genomes along with the *A. thaliana* genome. These protein sequences were then utilized to identify single-copy orthologous genes for the construction of evolutionary trees. The Orthofinder software (v.2.4.0) [32] was employed, with the parameters set as -M msa -T raxml. The resulting evolutionary trees generated by the software were subsequently edited and visualized using the online platform https://itol.embl.de/ (accessed on 21 September 2023).

### 2.3. Identification of Phosphorus-Utilization Genes in Fabaceae Crops

The 685 genes involved in PUE-related pathways (e.g., TCA cycle, glycolysis, CO_2_ fixation, and root development) from *A. thaliana* used in this study were published previously [22]. The protein sequences of 35 Fabaceae genes were compared with 685 phosphorus-utilization-pathway genes of *A. thaliana* using blastp software (v.2.10.1), and the filtering conditions were set as follows: Identity ≥ 35%, Coverage ≥ 50%, e-value ≤ 1 × 10^−20^.

### 2.4. Definition of the Amplification Modes of Homologous Genes

In the context of genomic evolution, there are three primary modes of gene amplification within genomes. The first is genes that possess syntenic relationships with any of the genomes of other Fabaceae plants, which are considered to have been inherited or amplified through whole-genome duplication. The second mode involves neighboring genes within a genome that exhibit sequence homology with each other, indicating that they have been generated through tandem duplication. The remaining repetitive genes, which neither were generated from whole-genome duplication nor belong to tandemly repeated genes, are categorized as the third type, referred to as dispersed repeated genes.

### 2.5. The Structure and Motif Prediction of Homologous Genes

The visualization of homologous gene structures was achieved using the online gene prediction tool GSDS v2.0 (https://gsds.gao-lab.org/ (accessed on 6 January 2024)). The prediction of motifs was conducted using the online software MEME (https://meme-suite.org/meme/ (accessed on 10 January 2024)), by providing the protein sequences of the genes and following the default parameter settings.

### 2.6. Analysis of Cis-Acting Element of RSL2 Genes

The *RSL2* promoter sequences consisting of 2000 bp sequences upstream of the transcription start site were obtained from the genomic sequence. An online tool for cis-regulatory analysis known as PlantCARE (https://bioinformatics.psb.ugent.be/webtools/plantcare/html/ (accessed on 25 January 2024)) was used to determine cis-acting regulatory elements.

### 2.7. Response of White Lupin to P Deficiency

We downloaded the RNA-seq data published previously to analyze the expression level of phosphorus-use-efficiency genes in this study [22]. In brief, leaves, stems, and roots of white lupin under −P and +P hydroponic solutions were harvested after 28 days of culture. The −P and +P nutrient solution was configured in the same way as described by Yan et al. [33]. Roots from the −P condition were further dissected into normal roots, PE (2–3 cm behind the root tip of first-order laterals), and cluster roots based on the developmental stages. Three biological replications were used for each experiment. The processes of mRNA extraction, sequencing, and gene expression level calculation followed the BGI standard methods. Differential expression analysis between −P and +P conditions was performed using DESeq package (v.1.10.1) in R (v.3.6.1) [34]. The resulting P values were adjusted using Benjamini and Hochberg’s approach for controlling the FDR. Genes with a |log2 fold-change| > 1 and FDR value < 0.01 were defined as differentially expressed. The expression dynamics of PUE genes in white lupin were visualized using the “ComplexHeatmap” R package (v.2.2.0) [35].

## 3. Results

### 3.1. Identification of Phosphorus-Use-Efficiency Gene Families in 35 Fabaceae Genomes

Fabaceae crops are rich in phenotypic variation and have a wide range of distribution worldwide. In addition to phenotypic variation, Fabaceae plants also exhibit diverse genomic changes, such as chromosome numbers, genome sizes, and gene numbers. The diversity of genomic variations in Fabaceae plants may contribute to their adaptability to different environments. 

**Figure 1:**
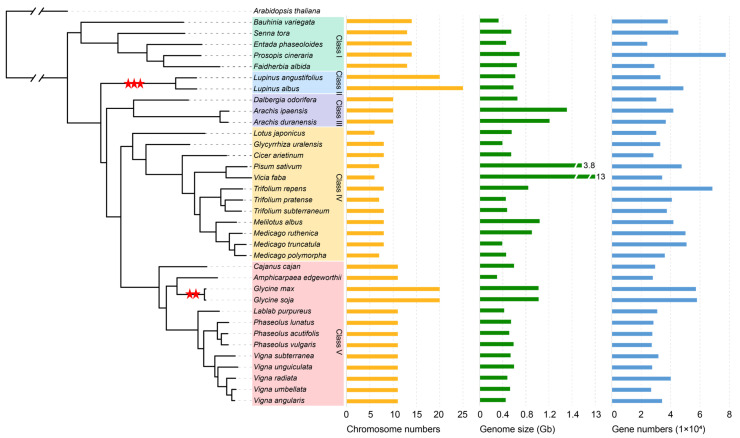
Genomic information and phylogenic tree of 35 Fabaceae genomes. The two red stars denote the whole-genome duplication event, while the three red stars denote the whole-genome triplication event.

Then phylogenic tree of 35 Fabaceae crops was constructed using single-copy orthologous genes with *A. thaliana* as an outgroup (Figure 1). These Fabaceae crops were divided into five classes, with woody plants of Fabaceae clustered in class I, which reflected that woody plants might be generated at an early stage. *L. albus* and *Lupinus angustifolius*, both belonging to the *Lupinus* genus, exhibited a close phylogenetic relationship and clustered together in class II, suggesting an earlier divergence time of the *Lupinus* genus. Additionally, *Dalbergia odorifera* and the two ancestral species of tetraploid peanut were clustered in class III. Other Fabaceae species clustered into two major classes. The class IV mainly consisted by the genera of *Trifolium* and *Medicago*, with several other genera in the outgroups, such as *Lotus*, *Glycyrrhiza*, *Cicer*, and *Pisum*. Class V was represented by the genera *Phaseolus* and *Vigna*, with the genera *Cajanus*, *Amphicarpaea*, and *Lablab* in the outgroup. In addition, *Glycine max* and *Glycine soja* were also in this class.

To further investigate the evolution and retention of phosphorus-use-efficiency (PUE) genes in Fabaceae plants, this study collected a total of 685 reported PUE genes in *A. thaliana*, encompassing 18 related biological pathways. These pathways include energy supply processes, such as glycolysis, the Calvin cycle, and the TCA cycle; root development processes involving the main root, lateral roots, and root hair development; enzyme secretion processes, like acid phosphatase secretion and ribonuclease secretion; and transport-related mechanisms for phosphate, organic acids, and sugars, etc. 

Then, the identification of PUE genes was analyzed in 35 Fabaceae genomes. Overall, an average of 1888 genes in the 35 Fabaceae genomes were found to be homologous with 685 Arabidopsis PUE genes (Table 1). There was an obviously expansion of the PUE pathway genes in Fabaceae plants, particularly in Glycine, where a total of 3014 PUE genes were identified, representing over four times that in Arabidopsis. Followed by Lupinus, the number of PUE genes in Lupinus was more than three times that in Arabidopsis. The Glycine genus exhibited the highest number of PUE genes, probably owing to its recent whole-genome duplication event that occurred approximately 8 to 13 million years ago [22,36]. This recent duplication event has allowed for the retention of a more intact set of duplicated genes. Conversely, Lupinus underwent a whole-genome triplication roughly 20 million years ago [22], which, through an extensive process of evolutionary selection, has resulted in the loss of numerous genes.

The number of PUE genes in other genera also exceeded twice that in Arabidopsis. Specifically, the most significant gene expansion was observed in PUE genes related to lateral root development, root hair development, proton transport, and other phosphorus-utilization pathways. The expansion of PUE gene pathways exhibited divergence among the genera of Fabaceae crops. For example, the number of genes expanded in proton transport and ribonuclease secretion pathways was the highest in Medicago, while the number of genes expanded in the TCA cycle for organic acid and acid phosphatase secretion was the highest in Lupinus. Although there were only two acid phosphatase secretion genes in *A. thaliana*, eight homologous genes were identified in Lupinus, far exceeding the number found in other genera, which reflected that this family was retained following polyploidy rather than being lost. This result indicated the significant expansion of acid phosphatase secretion genes in Lupinus, which was also reported in previous study [22].

### 3.2. Over-Retention of RSL2 Genes in L. albus

**Figure 2:**
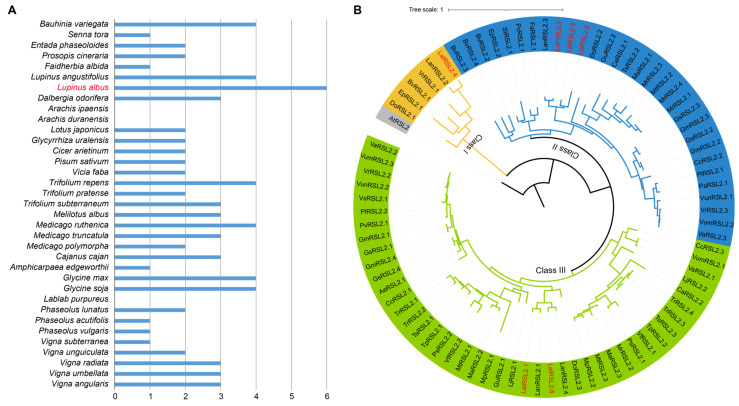
*RSL2* gene family numbers and polygenic tree in 35 Fabaceae genomes. (**A**) *RSL2* gene family numbers in 35 Fabaceae genomes. (**B**) Polygenic tree of *RSL2* gene family in 35 Fabaceae genomes. The six red genes came from white lupine.

*RSL2* is a gene in the root hair development pathway, and there have been studies reporting on its function in positively regulating root hair growth and development in *A. thaliana* [24]. In most Fabaceae crops, there are only one to four homologous genes of *RSL2*, with an average of two homolog genes in each genome. There are also some genomes that do not contain homologous genes of *RSL2*, such as the two diploid ancestors of peanuts (*Arachis duranensis* and *Arachis ipaensis*) and *Lablab purpureus*. However, six *RSL2* homologous genes were identified in the *L. albus* genome, which reflected that the *RSL2* gene exhibits specific expansion in white lupin (Figure 2A).

A total of 81 *RSL2* homologous genes were identified in 35 Fabaceae genomes. These *RSL2* genes could be divided into three classes in the polygenic tree constructed based on the protein sequence (Figure 2B). Among them, the number of gene families in class I was the smallest, including the homologous genes of some woody plants in Fabaceae and genes in the Lupine genus, which reflected that these genes may have been generated at the early stage. The other two classes contained 31 and 44 genes, respectively. Homologous genes from Medicago, Trifolium, Glycine, Phaseolus, and Vigna were distributed in both of the two classes, while the homologous genes in some crops were only distributed in only one class, such as *Pisum sativum*, *Vicia faba*, and *Lotus japonicus*, which indicated that some homologous genes in these genomes may have been lost during evolution. It is worth noting that the *RSL2* homologous genes of the Lupinus genus were distributed in all three classes. Moreover, there are three homologous genes of white lupin in class II, two of which were specific in white lupin, which may play an important role in the formation of cluster roots for environmental adaptations, such as the low-phosphorus response for white lupin.

### 3.3. RSL2 Genes Expanded through WGT in L. albus

**Figure 3:**
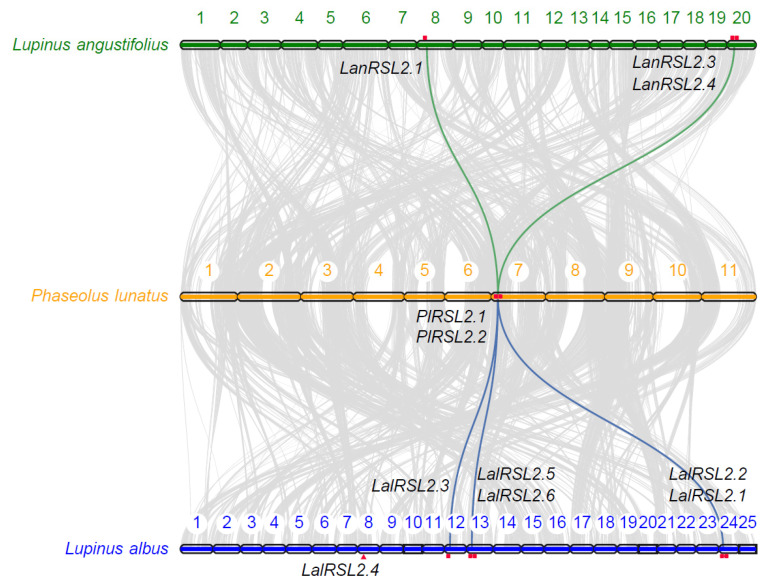
Expansion of *RSL2* gene family in *L. albus* and *Lupinus angustifolius*.

To investigate the influence of WGT on the amplification of *RSL2* homologous genes, we conducted syntenic alignment between *L. albus* and *Lupinus angustifolius* with *Phaseolus lunatus*, which have not experienced the WGT event (Figure 3). The homologous genes of *RSL2* in *P. lunatus* are two tandem duplicates, *PlRSL2.1* and *PlRSL2.2*, which are located on chromosome 7. Syntenic analysis revealed that these two tandem duplicated genes exist as two and three copies in *L. albus*, respectively, all of which were generated through WGT and located on chromosomes 12, 13, and 24. Specifically, the genes on chromosomes 13 and 24 still retain two tandem duplications, and only one copy exists on chromosome 12, suggesting the possible loss of another tandem duplicated gene during evolution. In the *Lupinus angustifolius* genome, only one or two copies of this tandem duplicated gene exist. One set of tandem duplications generated by WGT was completely lost, leaving only one set with two tandem duplications located on chromosome 20. These results reflected that the *RSL2* homologous genes generated through WGT have been more completely retained in the *L. albus* genome, potentially crucial for the adaptation of *L. albus* to variable environments, such as phosphorus deficiency.

### 3.4. LalRSL2s Exhibit a Diverse Gene Structure and Motif Composition 

**Figure 4:**
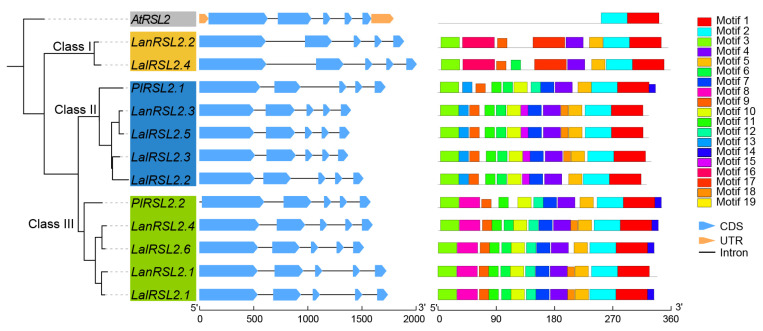
Gene structural and motif type analysis of *RSL2* gene family.

Further gene structure analysis of *RSL2* homologs in *L. albus* revealed that these genes exhibited differences in length, ranging from 1400 to 2000 bp (Figure 4). However, the exon lengths of these genes were relatively similar, with the primary length differences observed in introns. Among these, two genes with the longest lengths were at class I of the phylogenetic tree. The first exon lengths of these two genes were longer than those of other homologous genes, leading to an increase in gene length. In contrast, genes at class II and class III generally exhibited shorter intron lengths, potentially reflecting the result of evolutionary selection. It was observed that the gene lengths of *LalRSL2.3* and *LalRSL2.5* were shorter than other homologous genes in *L. albus*. 

Then, we analyzed the motif structures of these *RSL2* homologs. Although the gene length and exon number among Arabidopsis *RSL2* and Fabaceae homologs were similar, their motif types were significantly different. Most motifs were lost in the *AtRSL2* gene, leaving only motif 1 and 2. However, more motif numbers and types were observed in Fabaceae homologs, with motif 3, 4, and 5 being highly conserved in all Fabaceae homologous genes. These motifs might experience significant positive selection and may play a crucial role in the adaptation of Fabaceae crops to the environment. Consistent with the phylogenetic relationships, the motif types of Fabaceae homologs could also be clearly classified into three categories. Genes in Class I contained two conserved motifs16 and 17, which possessed a motif type distinct from the other two classes. Class II possessed two conserved motifs, 13 and 15, while Class III possessed two conserved motifs, 8 and 12. Among homolog genes of *L. albus*, genes *LalRSL2.3* and *LalRSL2.5* possessed the same motif structure and showed the shortest gene length, which indicated that these two genes might experience specific selection in *L. albus*.

### 3.5. LalRSL2s Contain Various Cis-Acting Elements in Promoter Regions 

**Figure 5:**
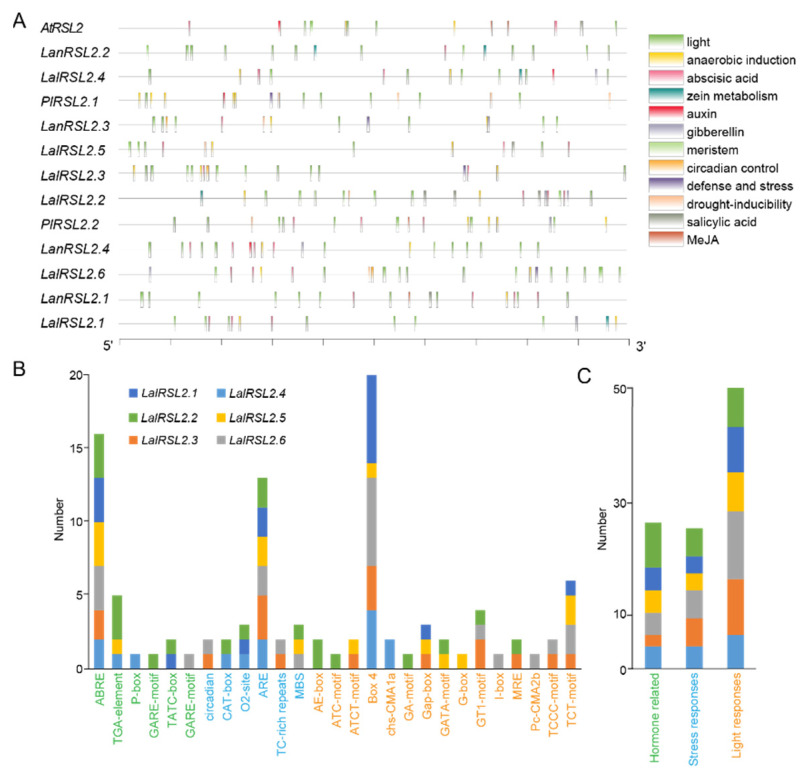
Analysis of cis-acting elements of *RSL2* gene family. (**A**) The main cis-acting elements in the promoter of *RSL2* genes. Differently colored boxes represent different cis-elements. (**B**) The number of different cis-acting elements and related responses (**C**) for six *RSL2* genes in *L. albus*.

The cis-acting elements in the gene promoter region could regulate gene expression levels by binding to transcription factors, affecting binding efficiency and stability. Thus, cis-acting elements were predicted in the 2000 bp region upstream of *RSL2* homolog genes (Figure 5). Using the PlantCARE website, a total of 15 types of cis-acting elements were identified, which are related to light-responsive, hormone-related, and stress-responsive processes. For *RSL2* homolog genes in *L. albus*, *Lupinus angustifolius* and *P. lunatus*, *LalRSL2.6* contained the largest number of cis-acting elements and then was *LalRSL2.2*, which indicated that these two genes of *L. albus* might generate gene functions or expression differentiation by containing more cis-acting elements in the promoter region. Notably, all *RSL2* family members in *L. albus* contained a cis-acting element involved in the abscisic acid responsiveness, and most of these genes contained a cis-acting element involved in the gibberellin or salicylic acid responsiveness, which suggests a potential link between the *RSL2* gene family and plant hormone signaling pathways.

The promoters of all *RSL2* family members contain a large number of light-responsive elements, such as the Box4, TCT-motif, GT1-motif, and Gap-box. Among them, the *LalRSL2.6* gene contained the most light-response elements, especially for Box4 elements. For hormone-related cis-acting elements, ABRE was the most enriched element in the promoter of *RSL2* family members in *L. albus*. The *LalRSL2.2* gene contained most hormone-related cis-acting elements, such as ABRE, TGA-element, GARE-motif, and TATC-box. For stress-response cis-acting elements, ARE was the most enriched element in the promoter. *LalRSL2.3* contained most stress-response cis-acting elements, such as ARE, TC-rich repeats, and circadian. These results reflected the diversity of cis-acting elements in the promoter of the *RSL2* family, which might suggests their potential role in stress responses and plant growth for *L. albus*.

### 3.6. LalRSL2s Exhibit Tissue-Specific Expression under Low-Phosphorus Stresses

**Figure 6:**
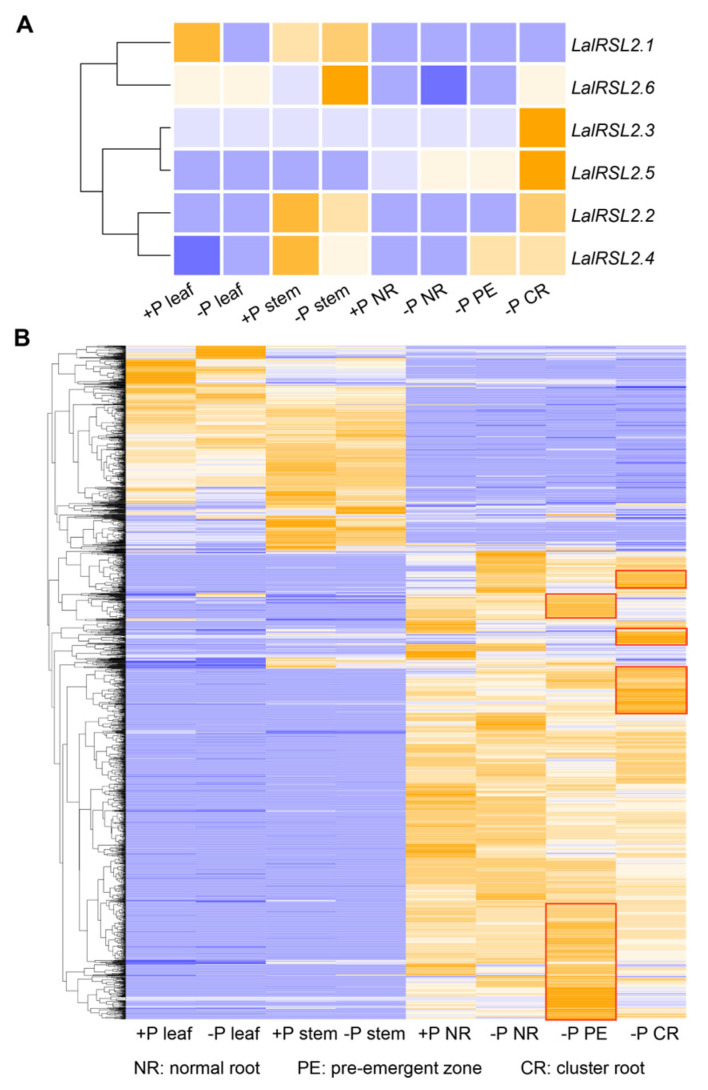
Expression heat map of PUE genes of white lupin under different treatments. (**A**) The expression of six *RSL2* genes and all PUE genes (**B**) of white lupin under different treatments. +P and −P represent the normal and low-phosphorus conditions, respectively. Genes in the red block were highly expressed in the pre-emergent zone or cluster root. The colors ranging from purple to orange represent the gene expression levels from low to high.

Transcriptome data of different tissues (leaves, stems, and roots) of *L. albus* under normal growth conditions and low-phosphorus treatment were collected for gene expression analysis. As shown in Figure 6A, there were differences in the expression patterns of six *RSL2* homologous genes in different plant tissues under different treatments. As mentioned above, *LalRSL2.2* and *LalRSL2.3* were specifically amplified genes unique to *L. albus* compared to *Lupinus angustifolius*. Both genes exhibited high expression levels in low-phosphorus cluster roots. Notably, *LalRSL2.3* exhibits specific and high-level expression exclusively in cluster roots, suggesting its potential important roles in the formation of cluster roots for *L. albus*. *LalRSL2.4* showed the highest expression level in stems and relatively high expression in low-phosphorus cluster roots and the pre-emergent zone of cluster roots. Additionally, *LalRSL2.5* also exhibited specific high expression in cluster roots. These four *RSL2* homologs might contribute to the formation of cluster roots in *L. albus* under low-phosphorus conditions. Specifically, *LalRSL2.6* was only highly expressed in low-phosphorus stems, while *LalRSL2.1* had the highest expression in leaves under normal growth conditions, in addition to relatively high expression in stems. These results indicate that the six *RSL2* homologs have functional divergence in *L. albus*, with four of them highly expressed in cluster roots and potentially involved in the formation of cluster roots during low-phosphorus adaptation in *L. albus*.

We also carried out an analysis of the expression of all PUE genes under different treatments. The expression heatmap showed that about 70% of the genes were highly expressed in roots, and a considerable number of genes were highly expressed in the cluster roots or pre-emergent zone of cluster roots (Figure 6B). These results also indicated that the PUE gene families might contribute to the formation of cluster roots and low-phosphorus adaptation in *L. albus*.

## 4. Discussion

Fabaceae plants possess high economic value, including woody plants such as *Bauhinia variegata*, *Faidherbia albida*, and *Prosopis cineraria*, oil-bearing crops like *Cajanus cajan* and *Arachis hypogaea*, miscellaneous grain crops like *Vigna angularis*, *Vigna radiata*, and *V. faba*, vegetable crops like *Phaseolus vulgaris*, *Vigna unguiculata*, and *P. sativum*, feed crops like *Trifolium pratense*, and *Medicago truncatula*, as well as medicinal plants like *Melilotus albus*, *D. odorifera*, and *Senna tora*. The morphological diversity within Fabaceae reflects their exceptional adaptability to diverse environmental conditions.

Phosphorus is an essential element for plant growth and development, which is absorbed in the form of phosphate. These fertilizers are predominantly extracted from non-renewable rock phosphate deposits; thus, it is imperative for agricultural productivity to maximize the efficient utilization of these finite resources. Plants adapt to phosphorus-deficient conditions through mechanisms, including organic acid and phosphatase secretion to dissolve soil-bound phosphorus, inducing high-affinity phosphate transporters, and reprogramming the root system architecture.

Whole-genome duplication events provide a substantial genetic reservoir in Fabaceae [2,6,37], facilitating the emergence of novel gene copies that contribute to morphological diversity and responses to environmental stimuli. All plant species have experienced polyploidization events [12], which are involved in plant speciation and the evolution of new functions. By identifying and characterizing gene families in various Fabaceae crops, we can gain a deeper understanding of the evolution of Fabaceae plants and the domestication selection of different gene families. The diploid soybean species have undergone two whole-genome duplication events, and the most recent event took place within the last 10 million years [38]. Following whole-genome duplication, soybean underwent a slow diploidization process, resulting in approximately 75% of its genes existing in multiple copies [39]. After the whole-genome duplication event in soybean, the remodeling of regulatory sequences led to the differentiation of transcriptional expression levels and tissue specificity of duplicated genes, thus generating diverse differentiation of gene functions [40]. White lupin has evolved from a WGT event, which makes it a great model to study the differentiation of gene functions for environmental adaptation [22].

White lupin has a remarkable ability to thrive in phosphorus-deficient soils through the development of the cluster root (CR) to improve the efficient utilization of phosphorus. The CR is a specialized structure with dense root hair that enhances phosphorus acquisition by increasing the surface area for phosphorus uptake and solubilizing phosphorus to make it more available for plant absorption. Molecular genetic studies have identified several key genes regulating root hair development [41,42,43]. Among them, the bHLH transcription factors ROOT HAIR DEFECTIVE 6 (RHD6), ROOT HAIR DEFECTIVE 6-LIKE 2 (RSL2), and ROOT HAIR DEFECTIVE 6- LIKE 4 (RSL4) appear to be crucial for hair morphogenesis [44]. Whereas *RHD6* is required for early stages of trichoblast development, *RSL4* appears to be the key gene regulating hair length [24,30,45]. *RSL2* has been shown to be responsive to phosphorus deficiency [46]. The root hair growth is initially triggered by the transcription factors (TFs) of the basic helix-loop-helix (bHLH) family RHD6 (ROOT HAIR DEFECTIVE 6)/RSL1 (ROOT HAIR DEFECTIVE 6 LIKE 1) in the initiation phase, and then, it is activated by the expression of *RSL4*/*RSL2* during the elongation phase [24,47]. Under high-phosphorus and high-auxin conditions, there is a direct effect of high phosphorus on the transcriptional regulation of *RSL2* to play an important role in root hair growth [48]. Mutants without root hairs show reduced inorganic orthophosphate uptake and compromised growth on soils when phosphorus availability is restricted. The constitutive expression of *RSL2* consistently improved phosphorus nutrition and increased phosphorus uptake per unit root length in *B. distachyon* [16]. Despite these impressive recent advances, the molecular mechanisms regulating root hair elongation under phosphorus stress remain to be uncovered.

In this study, we found that following whole-genome triplication, multiple copies of the *RSL2* gene were amplified and preserved in *L. albus*. Further transcriptome analysis revealed that under low-phosphorus treatment, most of the homologous *RSL2* genes in *L. albus* exhibited high expression levels in the cluster roots, which may play an important role in its functional differentiation and adaptation to low-phosphorus environments. Phosphorus was extracted from phosphate rock, representing a non-renewable resource, and its extensive application in agricultural fields often leads to environmental pollution. The specific phosphorus-utilization gene identified in this study from white lupin holds potential for functional validation through subsequent experiments. The unique low-phosphorus adaptation mechanism exhibited by white lupin can serve as a valuable reference for enhancing phosphorus use efficiency in plants. These insights provide a foundation for enhancing phosphorus utilization efficiency in crop breeding programs, contributing to the advancement of green agriculture and the conservation of phosphorus resources.

## 5. Conclusions

In summary, the present study is the first comprehensive analysis of PUE gene families across 35 Fabaceae species, uncovering a substantial expansion of PUE pathway genes within the Fabaceae family. This analysis revealed distinct patterns of amplification, evolution, and retention among various PUE pathways in different Fabaceae crops. Notably, the *L. albus* species exhibited a remarkably higher number of homologs of the root hair development gene *RSL2* compared to other Fabaceae species, which was attributed to the specific amplification and retention of multiple copies of the *RSL2* gene following WGT. We systematically explored the various characteristics of the *RSL2* gene in white lupin, including its phylogenetic relationship, gene structure, conserved motifs, and cis-elements, in addition to analyzing its specific expression pattern under different phosphorus conditions and multiple tissues. This study revealed a crucial role of the *RSL2* gene family in *L. albus* for developing cluster roots to adapt to a low-phosphorus environment.

## Figures and Tables

**Table 1:** Identification of phosphorus-utilization-related genes in Fabaceae.

PUE Pathways	Arabidopsis	Lupinus	Pisum	Trifolium	Medicago	Glycine	Phaseolus	Vigna	Fabaceae
Glycolysis	82	145	102	91	97	194	97	96	109
Calvin cycle	16	33	33	19	28	45	23	21	25
TCA cycle for organic acid	34	45	23	30	27	44	29	26	30
CO_2_ fixation in non-photosynthetic	20	50	42	31	42	64	36	39	40
Lipid remodeling	73	112	75	63	70	134	75	71	81
Primary root development	16	46	22	26	30	50	25	26	29
Lateral root development	21	83	65	65	65	139	66	71	73
Root hair development	50	264	188	182	211	371	218	212	226
Acid phosphatase	26	29	23	22	25	35	21	19	23
Secreted Acid phosphatase	2	8	3	4	3	5	3	3	4
Secreted ribonuclease	5	7	5	5	9	7	4	4	5
Phosphate uptake	9	12	10	7	11	14	9	8	10
Phosphate transport	25	35	30	23	25	48	24	25	28
Mitochondrion electron transport	10	23	19	16	19	31	19	18	19
Proton transport	24	115	78	103	120	111	94	89	90
Sugar transport	38	99	68	64	71	142	79	77	80
Organic acid transporter	70	73	78	59	75	129	69	70	76
Other PUE genes	164	1041	927	841	985	1453	902	853	940
Total number	685	2220	1791	1651	1913	3016	1793	1728	1888

## Data Availability

The original contributions presented in the study are included in the article, further inquiries can be directed to the corresponding author.
